# Safety, tolerability, pharmacokinetics and effects of diet on AD16, a novel neuroinflammatory inhibitor for Alzheimer’s disease: a randomized phase 1 study

**DOI:** 10.1186/s12916-023-03126-9

**Published:** 2023-11-23

**Authors:** Daizhuang Peng, Sumei Xu, Ting Zou, Yahui Wang, Wenjuan Ouyang, Yalan Zhang, Chengmei Dong, Dai Li, Jie Guo, Qiuying Shen, Xiaolei Hu, Wenzhi Zhou, Xiaomin Li, Qun Qin

**Affiliations:** 1 National Institution of Drug Clinical Trial, Xiangya Hospital, Central South University, Changsha, 410008 China; 2 National Clinical Research Center for Geriatric Disorders, Xiangya Hospital, Central South University, Changsha, 410008 China; 3 International Science and Technology Innovation Cooperation Base for Early Clinical Trials of Biological Agents in Hunan Province, Changsha, China; 4 Phase I Clinical Research Center, Xiangya Hospital, Central South University, Changsha, China; 5 The South China Center for Innovative Pharmaceuticals, Guangzhou, China; 6 Xiangya Changde Hospital, Central South University, Changde, China; 7 First People’s Hospital of Chongqing Liangjiang New Area, Chongqing, China

**Keywords:** Alzheimer’s disease, AD16, Safety, Tolerability, Pharmacokinetics, High-fat diet

## Abstract

**Background:**

AD16 is a Class 1.1 new drug candidate for Alzheimer’s disease (AD), which has demonstrated potential benefits in AD by reducing neuroinflammation in preclinical studies. Herein, the pharmacokinetics (PK), safety, and tolerability of single and multiple-dose AD16 and the effect of food were assessed in healthy Chinese adults.

**Methods:**

Single-center, randomized, placebo-controlled, double-blind studies were conducted for single and multiple ascending doses. A total of 62 subjects were enrolled in single-dose groups; 10 each in 5, 10, 20, 30, and 40 mg groups, and 6 each in 60 and 80 mg dose groups. Twenty subjects were divided equally into 30 and 40 mg groups for the multiple-dose study. To determine the effect of a high-fat diet on AD16, 16 subjects were administered a single 20 mg dose of AD16 under the fasted and fed condition in a single-center, randomized, open-label, two-cycle, two-crossover study. Moreover, safety and PK parameters were also assessed.

**Results:**

Plasma exposure to a single oral dose of AD16 increased at an approximate dose-increasing rate. The pharmacodynamic dose of the AD16 can be maintained through the accumulation effect of the drug within the safety window. Compared to fasting, ingesting a high-fat meal decelerated the rate of AD16 absorption, albeit without effect on its overall absorption. No dose-related toxicities were seen in any of the studies, all treatment-emergent adverse events were grade I/II, and no serious adverse event occurred.

**Conclusions:**

The present study exhibited favorable safety, tolerability, and PK profile of AD16, supporting its further research as a potential drug treatment for AD.

**Trial registration:**

ClinicalTrials.gov; NCT05787028, NCT05787041, NCT05806177. The SAD and FE studies were retrospectively registered on 28 March 2023. The MAD study was retrospectively registered on 10 April 2023.

**Supplementary Information:**

The online version contains supplementary material available at 10.1186/s12916-023-03126-9.

## Background

Alzheimer’s disease (AD) is a progressive, chronic, irreversible, and incurable brain condition characterized by declining cognitive ability and increasing functional disability [1, 2]. Since AD commonly develops in elderly people and severely threatens their health, its incidence and consequent health burden are expected to escalate as the global population ages [3, 4]. Current therapeutic options for AD mostly focus on providing symptomatic relief and delaying its progression [5]. However, specific measures to cure and prevent AD are not yet available [6]. This lack of treatment for AD creates a substantial unmet medical need and draws the attention of researchers worldwide to develop new treatment entities.

Although the pathophysiological mechanism of AD is complex and not yet fully understood, its pathological features are principally attributed to the deposition of extracellular amyloid beta (Aβ) peptide that constitutes senile plaques (SP), formation of tau protein-associated neurofibrillary tangles (NFTs), and loss of synapses [7–11]. Considering these hallmarks of AD, most of the recent attempts to develop new medicines have been directed toward Aβ peptide and NFTs. Unfortunately, these new medications faced unusual rates of clinical failures [12–14]; AD clinical trials accomplished a success rate of only 0.4% from 2002 to 2012 [15]. Consequently, the development of disease-modifying drugs is immensely significant.

Some studies have shown that microglia play an important role in the pathogenesis of AD by releasing inflammatory mediators such as inflammatory cytokines, complement, chemokines, and free radicals [16]. Microglia neuroinflammation is a prominent feature of AD, possibly with a crucial role in disease development [17, 18]. Thus, targeting neuroinflammation by inhibiting the microglia overactivation and the release of pro-inflammatory cytokines may be an effective strategy to prevent the worsening of AD.

**Fig. 1:**
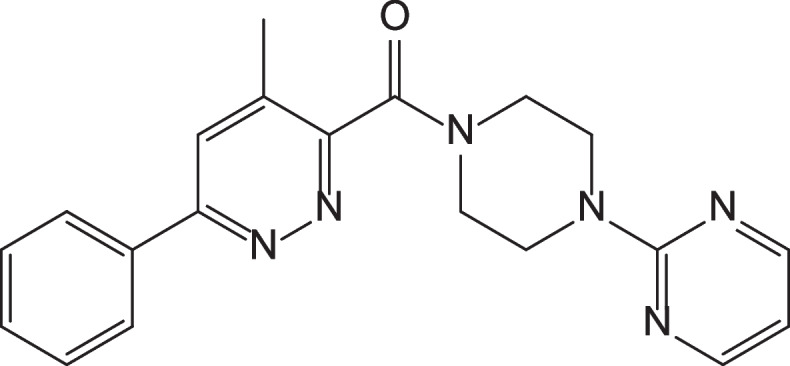
Chemical structure of AD16

Taking this into account, the Guangzhou Institute of Biomedicine and Health, Chinese Academy of Sciences, and the South China Center for Innovative Pharmaceuticals collaboratively developed a new neuroinflammation inhibitor AD16, also known as (2-pyrimidinepiperazinyl)-(4-methyl-6-phenylpyridazine)-methanone (Fig. 1). It is a Class 1.1 new drug candidate for AD. Regulating microglial activation/senescence and restoring physiological functions by enhancing lysosomal function are two potential mechanisms of AD16 [19]. Preclinical studies showed that AD16 could inhibit brain interleukin-1, reduce the induced activation of glial cells, and block Aβ peptide production in transgenic and other traditional animal models of AD. The animal efficacy test of AD16 is comparable to that of donepezil and memantine [20]. In addition, AD16 was detected in rat hippocampus, cerebral cortex, and other brain tissues, suggesting that AD16 can successfully cross the blood–brain barrier. Acute toxicity test showed a maximum tolerated dose (MTD) of 2000 mg/kg in rats and dogs and an effective dose of 0.0025–0.25 mg/kg in mice. Preclinical pharmacodynamic experiments have proved that AD16 has obvious effect in the treatment of AD, clear target, large safety window, and druggability is superior to similar developed varieties abroad. Furthermore, AD16 protects against oxygen–glucose deprivation-induced astrocytes, neuronal cell damage, and hypoxia–ischemia-induced brain damage [21].

The present study aimed to assess the pharmacokinetics (PK), safety, and tolerability of AD16 in healthy Chinese adults after single and multiple ascending doses. Moreover, the effect of a high-fat diet on the PK parameters of AD16 was investigated.

## Methods

We used the CONSORT reporting guidelines [22]. CONSORT checklist is available in Additional file 1.

### Study design and conduct

The present study was conducted in three arms: single ascending dose (SAD), multiple ascending dose (MAD), and food effect (FE) arm. Single-center, randomized, placebo-controlled, double-blind studies were conducted for SAD and MAD. The subjects, researchers, and monitors were not aware of the drug dispensation. The placebo is similar in shape and weight to the trial drug.

#### SAD

Following a preliminary study, 70 subjects were enrolled in the SAD arm. Among these, eight subjects underwent pretesting, while 62 took the formal trial. In the SAD study, AD16 was tested in a total of seven dose groups: 5, 10, 20, 30, 40, 60, and 80 mg. Ten subjects were included in each of the first five groups; eight received the trial drug, and two received a placebo. Six subjects were enrolled in each of the 60 and 80 mg dose groups; 4 received the trial drug, and 2 received a placebo. The 80 mg group was only studied for safety and tolerance, and PK was not studied. Urine and fecal samples were collected in the 20 mg dose group for the mass balance study. The next dose was administered after determining the safety of the prior dose.

#### MAD

This study arm was a multiple-dose escalation trial of AD16 in the 30 mg (bid) and 40 mg (bid) groups, each consisting of 10 subjects. The trial drug and placebo were administered to eight and two subjects twice a day, respectively, in each group from day 3 to day 8. This study aimed to assess the safety, tolerability, and PK features of AD16 in healthy adults following its repeated administrations.

#### FE

This study was designed as a single-center, randomized, open-label, two-cycle, two-crossover, single-dose food effect study to evaluate the effects of a high-fat diet on the PK and safety of AD16 tablets in healthy adults. Assessing the effects of food on drug bioavailability is important to ensure the safety and efficacy of drugs for clinical use. The study evaluated the situation and degree of drug exposure in the body by comparing PK parameters under the conditions of fasting and eating a high-fat diet, so as to evaluate whether food has an influence on AD16. A total of 16 subjects were randomly divided into two equal groups: A and B. According to the group, the subjects received a single dose of AD16 at cycles 1 and 2 under fasted state or after consuming a standardized high-fat meal comprising a total calorie count of 916, including 168, 499, and 249 cal of proteins, fats, and carbohydrates, respectively.

### Subjects

**Fig. 2:**
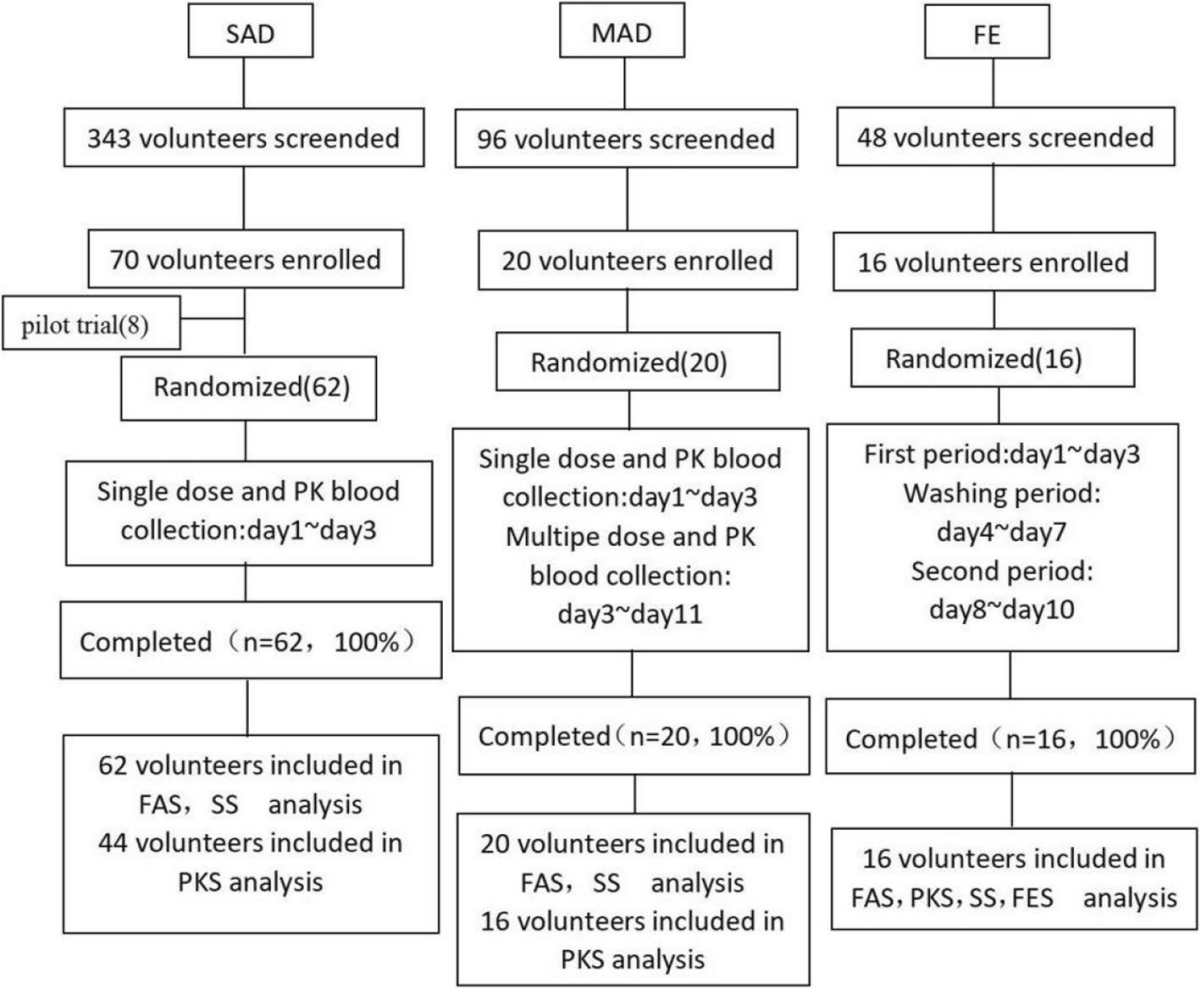
Flow chart of study. Forty-four volunteers received AD16 in the 5 mg to 60 mg dose group of SAD. FAS, full analysis set; PKS, PK analysis set; SS, safety analysis set; FES, FE analysis set

Subjects were recruited through recruitment advertisements. A total of 487 healthy subjects from various communities in China underwent screening; 343 for SAD, 96 for MAD, and 48 for FE arm (Fig. 2).

The eligible population consisted of healthy Chinese males and females weighing ≥ 50 kg or ≥ 45 kg, respectively, aged 18–45 years, and having a body mass index (BMI) of 19–24 kg/m^2^. The exclusion criteria included infectious or serious diseases; allergy to components analogous to the trial drug; history of frequent nausea, vomiting, or drug abuse; drug-positive urine test during the screening period; noncompliance to diet provided by the clinical research center; those who smoked more than or equal to 5 cigarettes per day in the 3 months before the trial; and positive breath test for alcohol. Detailed inclusion and exclusion criteria are available in Additional file 2: Supplementary information S1.

### PK evaluation

Whole blood samples were collected at a phase I clinical research center. Blood samples were taken at the following time points:

- SAD and FE—pre-dose and 0.083, 0.167, 0.25, 0.5, 1, 2, 3, 4, 6, 8, 12, 24, and 48 h post-dose.
- MAD—days 1 and 9: pre-dose and 0.083, 0.167, 0.25, 0.5, 1, 2, 3, 4, 6, 8, 12, 24, and 48 h post-dose; days 6, 7, and 8: pre-morning dose.

Liquid chromatography-tandem mass spectrometry (LC–MS/MS) was used for the determination and semi-quantitative analysis of AD16 and its possible metabolites in plasma, urine, and feces.

Various PK parameters were evaluated, including time to reach peak plasma concertation (*t*_max_), maximum plasma drug concentration (*C*_max_), elimination half-life (*t*_1/2_), area under the plasma concentration–time curve (AUC) from time zero to infinity (AUC_0-∞_) and from time zero to *t* (AUC_0-t_), apparent total clearance (CL/F) and apparent volume of distribution (Vd/F) after oral administration, mean retention time (MRT) from the first dosage to infinity (MRT_0-t_) and from time zero to *t* hours (MRT_0-t_), terminal disposition rate constant/terminal rate constant (λ_z_), accumulation ratio (*R*_ac_), and degree of fluctuation (DF), the cumulative amount of unchanged drug excreted in urine/feces (Ae), cumulative rate of drug excretion through urine/feces (Fe_0-*t*_), and renal clearance of drug from plasma (CLr).

### Safety and tolerability assessment

The safety and tolerability of AD16 were evaluated by recording the incidence and severity of adverse events during the clinical study, the incidence of serious adverse events (SAE), changes in clinical laboratory results (routine blood, biochemistry, coagulation, and urine routine), clinical symptoms, vital sign measurements, 12-lead electrocardiogram (ECG), and physical examination. Adverse events were coded using the Chinese version of MedDRA 23.0. The severity of adverse events was determined according to the Common Terminology Criteria for Adverse Events (CTCAE 5.0). The associations between adverse events and drugs were expressed in five categories: definitely, probably, possibly, unlikely related, and unrelated. During the dose escalation phase, the subjects’ intolerance was considered if more than half of the participants experienced a grade II or higher drug-related adverse event, or more than a quarter of participants suffered from grade III-IV drug-related adverse events, or upon occurrence of one drug-related SAE.

### Statistical analysis

The sample size for SAD and MAD studies adhered to the “Guidelines for clinical pharmacokinetic studies of chemical drugs” by the China National Medical Products Administration, which recommends 8–12 healthy subjects in each dose group for both studies. However, in order to reduce drug exposure in high-dose groups for safety and tolerability reasons, the 60 mg and 80 mg groups for the SAD study were limited to 6 subjects per group. The sample size for the FE study was determined according to the “Food-Effect Bioavailability and Fed Bioequivalence Studies” by United States Food and Drug Administration, which recommends enrolling at least 12 subjects in the FE group. No formal statistical calculation was used to predefine the sample size. The study administrator used SAS version 9.4 of the PLAN program to allocate study subjects randomly. Randomized tables for each dose group in SAD and MAD studies were generated with the ratio of AD16/placebo. The FE arm was randomly divided into two groups in a 1:1 ratio using a stratified block randomization method. Further details about study randomization are provided in Additional file 2: Supplementary information S2.

Full analysis set (FAS) consisted of all subjects who underwent randomization and received at least one dose. All randomized subjects with at least one dose and post-dose safety information were included in the safety analysis set (SS). All subjects who underwent randomization and had at least one measurable concentration without a protocol deviation that could have impacted the PK data are included in the PK analysis set (PKS).

PK parameters in PKS were computed using the WinNonlin version 6.4 non-AV model. The relationship between drug dose and drug plasma exposure was assessed by plotting the scatterplots of log-transformed PK parameters (*C*_max_, AUC_0-*t*_, and AUC_0-∞_) related to the log dose. The proportional dose–response relationship of AD16 tablets following a single fasting administration was assessed using a power model (PK parameter = (dose)). Following natural logarithm transformation, Log (PK parameter) = log (*α*) + *β* log (dose) + *ε* was used as the model, taking the intercept as *α* and the slope as *β*. In order to evaluate the linearity, the overall slope was estimated using 90% confidence intervals (CI), and the linear relationship was estimated using *β*.

The impact of taking food on AD16 tablets was assessed by calculating the geometric mean ratio (GMR) of fasting and post-prandial AUC_0-*t*_, AUC_0-∞_, and *C*_max_ ratio and its 90% CI utilizing a linear mixed-effects model. Treatment (fasted vs fed), time, and grouping were considered fixed effects, and subjects (dosing order) were treated as random effects.

Statistical analysis was performed using the statistical software SAS 9.4. AD16 plasma, urine, and fecal concentrations were tabulated, and descriptive statistical analyses were conducted by dose group, subject, and visit/sampling time point. The research results were reported in the form of summary tables and lists.

## Results

### Demographics and baseline characteristics

The FAS set comprised 62 members from the SAD group, 20 from MAD, and 16 from FE. The groups in three arms presented fairly even distribution of age, gender, height, weight, and BMI. The baseline characteristics and demographics of study participants are summarized in Table 1.

**Table 1:** Demographics and baseline characteristics (FAS)

	SAD group								MAD group			FE group	
	Placebo (*n* = 14)	5 mg (*n* = 8)	10 mg (*n* = 8)	20 mg (*n* = 8)	30 mg (*n* = 8)	40 mg (*n* = 8)	60 mg (*n* = 4)	80 mg (*n* = 4)	Placebo (*n* = 4)	30 mg (*n* = 8)	40 mg (*n* = 8)	A (*n* = 8)	B (*n* = 8)
Age													
Mean (SD)	22.7 (3.0)	23.8 (5.3)	23.4 (4.2)	22.0 (4.0)	21.4 (3.5)	22.9 (3.0)	26.5 (5.7)	22.3 (5.2)	21.3 (3.0)	23.5 (2.1)	24.5 (6.2)	23.9 (3.2)	23.9 (4.0)
Median (range)	22.5 (19–28)	22.0 (19–34)	22.0 (19–32)	21.5 (18–31)	20.0 (19–29)	23.5 (18–26)	26.5 (20–33)	20.0 (19–30)	21.0 (18–25)	23.5 (21–26)	21.5 (18–35)	23.0 (19–28)	24.5 (19–29)
Sex (%)													
Male	8 (57.1)	5 (62.5)	5 (62.5)	4 (50.0)	5 (62.5)	5 (62.5)	2 (50.0)	3 (75.0)	2 (50.0)	5 (62.5)	4 (50.0)	4 (50.0)	4 (50.0)
Female	6 (42.9)	3 (37.5)	3 (37.5)	4 (50.0)	3 (37.5)	3 (37.5)	2 (50.0)	1 (25.0)	2 (50.0)	3 (37.5)	4 (50.0)	4 (50.0)	4 (50.0)
Height													
Mean (SD)	163.5 (7.6)	162.1 (4.4)	167.7 (9.8)	161.7 (9.4)	162.0 (8.6)	166.0 (6.6)	164.4 (9.1)	169.9 (7.8)	164.8 (5.8)	167.6 (9.9)	163.7 (13.7)	162.8 (8.8)	165.0 (7.1)
Median (range)	163.3 (148.0–175.5)	161.8 (153.5–168.0)	167.5 (151.0–183.0)	158.8 (150.0–176.0)	162.0 (151.5–178.0)	165.3 (157.0–176.0)	161.8 (156.5–177.5)	169.3 (163.0–178.0)	165.3 (157.5–171.0)	169.5 (150.5–180.0)	168.3 (146.0–185.5)	164.0 (151.0–174.5)	165.8 (151.5–175.5)
Weight													
Mean (SD)	57.5 (7.5)	57.0 (3.6)	59.6 (4.1)	53.9 (7.2)	55.9 (5.7)	59.3 (7.5)	56.8 (5.7)	57.9 (4.0)	59.7 (3.9)	58.9 (7.1)	56.6 (9.4)	56.3 (7.0)	57.4 (8.3)
Median (range)	55.9 (46.4–71.9)	56.9 (52.3–63.8)	59.7 (51.1–64.5)	52.0 (45.4–66.0)	56.0 (46.0–65.0)	58.7 (49.2–69.4)	54.7 (52.6–65.3)	58.9 (52.4–61.3)	58.8 (55.9–65.1)	58.6 (49.2–67.4)	55.4 (47.6–76.7)	54.8 (49.0–69.2)	55.9 (48.0–71.2)
BMI													
Mean (SD)	21.4 (1.4)	21.7 (1.2)	21.2 (1.4)	20.5 (0.6)	21.3 (1.3)	21.5 (1.5)	21.0 (1.0)	20.1 (0.9)	22.0 (1.7)	20.9 (1.5)	21.0 (1.2)	21.2 (1.2)	21.0 (1.7)
Median (range)	21.40 (19.2–23.7)	21.80 (19.5–23.4)	21.35 (19.3–23.3)	20.30 (19.8–21.3)	21.25 (19.3–23.7)	21.10 (19.3–23.7)	20.85 (20.0–22.3)	19.75 (19.3–21.4)	22.10 (20.2–23.6)	20.60 (19.3–23.2)	21.30 (19.4–22.3)	21.05 (19.8–23.8)	20.85 (19.0–23.7)

The units of height, weight and BMI are cm, kg, kg/m^2^

### SAD PK

The PK results in the SAD arm showed a median peak time of 0.5–1.0 h with median *t*_1/2_ ranging from 5.54 to 6.94 h. The *C*_max_ and AUC increased with an increase in the proportion of dosage. However, this incremental trend was less prominent in exposure to the drug at 40 and 60 mg doses (Table 2).

**Table 2:** PK parameters of AD16 in SAD group

PK parameters		5 mg *N* = 8	10 mg *N* = 8	20 mg *N* = 8	30 mg *N* = 8	40 mg *N* = 8	60 mg *N* = 4
*T*_max_ (h)	Median (range)	0.50 (0.25–2.00)	0.50 (0.25–2.00)	1.00 (0.33–2.00)	0.75 (0.33–2.00)	0.50 (0.33–1.00)	0.87 (0.50–1.00)
T_1/2z_ (h)	Mean ± SD (CV%)	6.46 ± 2.05 (31.80)	6.51 ± 1.16 (17.90)	6.34 ± 1.32 (20.88)	6.43 ± 1.46 (22.77)	6.47 ± 1.38 (21.42)	5.85 ± 1.20 (20.48)
	Median (range)	5.42 (4.70–10.44)	6.38 (4.77–8.35)	6.58 (4.67–8.19)	6.27 (4.64–9.02)	6.94 (4.06–7.93)	5.54 (4.76–7.55)
*C*_max_ (ng/mL)	Mean ± SD (CV%)	181.6 ± 52.17 (28.73)	324.8 ± 67.70 (20.85)	582.0 ± 190.06 (32.66)	823.1 ± 164.87 (20.03)	1139.9 ± 174.69 (15.32)	1392.5 ± 404.34 (29.04)
	Median (range)	209.50 (111–236)	355.50 (224–389)	511.50 (411–943)	856.00 (564–1000)	1190.00 (913–1420)	1385.00 (1010–1790)
AUC_0-t_ (h*ng/mL)	Mean ± SD (CV%)	974.23 ± 227.30 (23.33)	1876.42 ± 443.64 (23.64)	3991.79 ± 962.47 (24.11)	5672.23 ± 1715.99 (30.25)	6808.53 ± 2167.28 (31.83)	9726.83 ± 1577.54 (16.22)
	Median (range)	934.84 (745.97–1294.06)	1820.15 (1302.29–2741.21)	3812.11 (2742.19–5444.71)	5193.31 (3921.47–8449.84)	6154.08 (4077.52–10,528.26)	9455.22 (8108.47–11,888.39)
AUC_0-∞_ (h*ng/mL)	Mean ± SD (CV%)	999.52 ± 229.69 (22.98)	1942.97 ± 439.23 (22.61)	4056.10 ± 948.02 (23.37)	5772.89 ± 1715.49 (29.72)	6881.45 ± 2184.89 (31.75)	9771.35 ± 1632.07 (16.70)
	Median (range)	965.56 (763.69–1317.42)	1883.37 (1337.48 –2789.17)	3833.06 (2817.34–5524.11)	5274.72 (4021.40–8646.43)	6186.26 (4129.88–10,620.99)	9472.10 (8121.11–12,020.10)
CL/F (mL/h)	Mean ± SD (CV%)	5231.05 ± 1148.84 (21.96)	5371.31 ± 1173.65 (21.85)	5166.19 ± 1174.41 (22.73)	5587.49 ± 1531.42 (27.41)	6336.59 ± 1956.32 (30.87)	6262.70 ± 984.38 (15.72)
	Median (range)	5201.73 (3795.29–6547.13)	5321.49 (3585.30–7476.74)	5217.85 (3620.49–7098.89)	5760.56 (3469.64–7460.09)	6558.11 (3766.13–9685.51)	6335.51 (4991.64–7388.16)
V_d_/F (mL)	Mean ± SD (CV%)	46,201.74 ± 6459.12 (13.98)	48,804.92 ± 3816.97 (7.82)	45,467.84 ± 4745.20 (10.44)	49,491.16 ± 7854.11 (15.87)	56,108.07 ± 9536.99 (17.00)	51,801.38 ± 5651.42 (10.91)
	Median (range)	45,646.41 (38,311.17–57,183.13)	48,397.36 (43,203.97–54,977.22)	47,202.23 (38,391.01–50,999.27)	47,556.53 (37,375.58–60,281.43)	55,361.62 (38,758.73–71,727.88)	52,900.10 (44,111.04–57,292.49)
MRT_0-t_ (h)	Mean ± SD (CV%)	7.02 ± 2.62 (37.32)	7.07 ± 1.68 (23.70)	7.71 ± 1.63 (21.10)	7.73 ± 1.84 (23.76)	7.49 ± 2.10 (28.03)	7.96 ± 1.66 (20.83)
	Median (range)	5.65 (4.82–11.04)	6.37 (5.4–10.18)	7.95 (5.72–9.85)	7.96 (5.15–10.68)	7.49 (4.39–10.20)	7.81 (6.45–10.53)

**Fig. 3:**
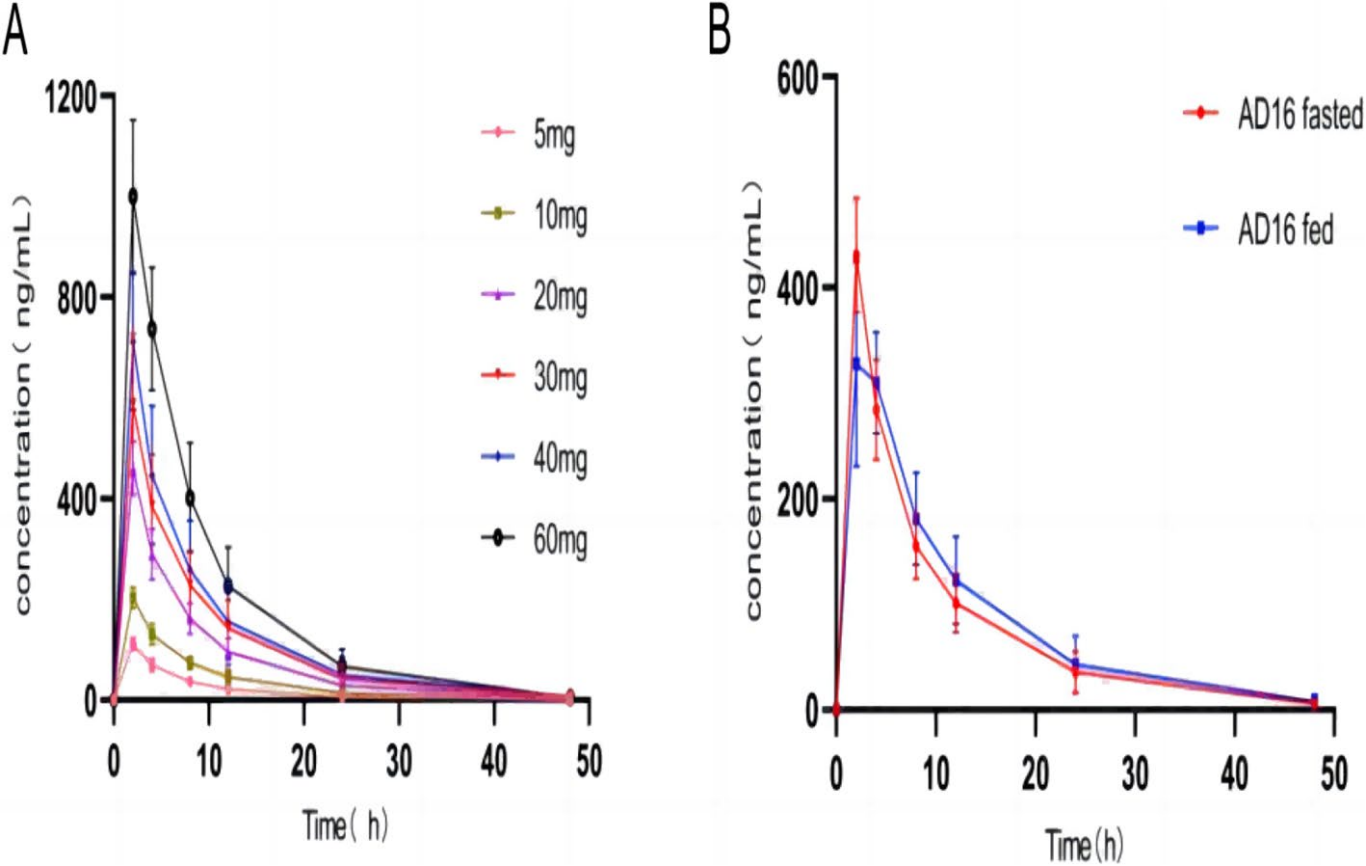
Mean blood concentration–time curves of AD16 in **A** SAD and **B** FE groups. Different dosage groups of SAD received a single administration of AD16 tablets. FE study subjects received 20 mg AD16 either in fasted or fed state

The *β* values (90% CI) of *C*_max_, AUC_0-*t*_, and AUC_0-∞_ and dose linear analysis were 0.86 (0.78–0.97), 0.94 (0.86, 1.02), and 0.93 (0.85, 1.01), respectively, in the dosage range of 5 mg to 60 mg. The *β* values of the three were close to 1, but 90% CI of them did not completely fall within the judgment interval, so the conclusion of whether they had linear pharmacokinetic characteristics was unclear. The average blood-time distribution of different dose groups is shown in Fig. 3, from which it can be seen that the blood concentration of AD16 increased with the dose increase.

### MAD PK

The statistical analysis of the PK parameters in the MAD arm (Table 3) revealed higher *C*_max_ and in vivo drug exposure in the 40 mg than 30 mg group. A comparatively lower degree of fluctuation in the 40 mg group indicated its higher stability than the 30 mg group. The *R*_ac_ of the 30 mg and 40 mg groups was 1.36 ± 0.24 and 1.68 ± 0.19, respectively, which demonstrated that the degree of quantitative accumulation for the two doses increased with a roughly linear dose ratio. In the steady state, when the dose was increased from 30 to 40 mg, *C*_max,ss_ increased at approximate dose ratios (1127.38 ± 205.57 to 1528.75 ± 320.96), and the AUC_0-τ,ss_ of 40 mg group increased to 1.6 times that of 30 mg group (30 mg and 40 mg were 5743.12 ± 1207.85 and 9395.79 ± 1533.46, respectively).

**Table 3:** PK parameters of AD16 in MAD groups

PK parameters	30 mg *N* = 8	40 mg *N* = 8
*T*_max,ss_ (h)	0.62 (0.33–1.00)	0.88 (0.50–4.00)
T_1/2,ss_ (h)	7.24 ± 1.35 (18.69)	8.50 ± 1.63 (19.15)
*C*_max,ss_ (ng/mL)	1127.38 ± 205.57 (18.23)	1528.75 ± 320.96 (20.99)
*C*_avg,ss_ (ng/mL)	478.59 ± 100.65 (21.03)	782.98 ± 127.79 (16.32)
*C*_min,ss_ (ng/mL)	204.88 ± 73.91 (36.08)	449.63 ± 85.91 (19.11)
AUC_0-τ,ss_ (h*ng/mL)	5743.12 ± 1207.85 (21.03)	9395.79 ± 1533.46 (16.32)
AUC_0-48 h,ss_ (h*ng/mL)	8282.70 ± 2266.75 (27.37)	14,737.31 ± 3178.18 (21.57)
AUC_0-∞,ss_ (h*ng/mL)	8377.29 ± 2338.26 (27.91)	15,061.14 ± 3425.91 (22.75)
CL/F_,ss_ (mL/h)	5444.94 ± 1210.19 (22.23)	4353.55 ± 679.42 (15.61)
V_d_/F_,ss_ (mL)	55,625.89 ± 10,662.74 (19.17)	52,688.80 ± 9545.88 (18.12)
*R*_ac,ss_ (%)	1.36 ± 0.24 (17.81)	1.68 ± 0.19 (11.12)
DF (%)	198.09 ± 50.69 (25.59)	137.29 ± 23.01 (16.76)

Data are expressed as mean ± SD (CV%) unless otherwise specified. *T*_max,ss_ (h) is expressed as median (range)

### The effect of food on PK of AD16

The arithmetic mean of λ_z_, *t*_1/2_, MRT_0-*t*_, MRT_0-∞_, CL/F, and Vd/F showed approximately similar characteristics for the elimination and distribution of AD16 tablets under fasting and fat-rich diet-fed states (Table 4).

**Table 4:** PK parameters of AD16 (20 mg) in FE arm

PK parameters	Fasted (*N* = 16)	Fed (*N* = 16)
*T*_max_ (h)	0.87 (0.50–2.00)	2.00 (0.50–4.00)
T_1/2z_ (h)	7.41 ± 2.02 (27.23)	7.19 ± 2.02 (28.04)
*C*_max_ (ng/mL)	560.60 ± 124.96 (22.29)	403.10 ± 89.63 (22.23)
AUC_0-t_ (h*ng/mL)	4143.77 ± 931.63 (22.48)	4154.41 ± 1286.46 (30.97)
AUC_0-∞_ (h*ng/mL)	4224.51 ± 981.28 (23.23)	4263.01 ± 1338.75 (31.40)
AUC__%Extrap_ (%)	1.81 ± 1.57 (84.94)	2.56 ± 1.70 (66.69)
CL/F (mL/h)	4976.05 ± 1139.73 (22.90)	5153.49 ± 1683.51 (32.67)
Vd/F (mL)	50,548.34 ± 6031.25 (11.93)	49,778.22 ± 7718.92 (15.51)
λ_z_ (1/h)	0.10 ± 0.03 (25.31)	0.10 ± 0.03 (25.66)
MRT_0-t_ (h)	8.64 ± 1.94 (22.44)	9.65 ± 2.41 (24.99)
MRT_0-∞_ (h)	9.41 ± 2.40 (25.52)	10.61 ± 2.70 (25.44)

Data are expressed as mean ± SD (CV%) unless otherwise specified. *T*_max_ (h) is expressed as median (range)

Under post-prandial administration conditions, *C*_max_ was lower, and *T*_max_ was longer than fasting administration (Table 4). The 90% CI of GMR of AUC_0-*t*_ and AUC_0-∞_ under fasted and fed states were between 80.00% and 125.00%, satisfying the evaluation criteria of bioequivalence. However, the 90% CI of the GMR of *C*_max_ did not fall within the range of 80.00% ~ 125.00%, failing to meet the bioequivalence evaluation criteria [*C*_max_: GMR (71.91), 90% CI (64.31, 80.42); AUC_0-*t*_: GMR (98.03), 90% CI (88.41, 108.71); AUC_0-∞_: GMR (98.78), 90% CI (88.98, 109.67)]. According to the average blood concentration (± SD)-time distribution of different treatments (fasted VS fed) (Fig. 3), a high-fat diet significantly reduced the *C*_max_ of AD16. In conclusion, a high-fat diet has a significant effect on *C*_max_ and can significantly reduce its absorption rate without any significant effect on its AUC.

### Metabolic transformation

Five metabolites of AD16 were identified in plasma, urine, and fecal samples, which were speculated to be mainly mediated by oxidation, hydrolysis, N-dealkylation, glucuronic acid binding, and sulfonic acid reaction. After oral administration of AD16 tablets, little AD16 was excreted in urine and feces. Cumulative excretion of AD16 in urine accounted for 0.17% of the administered dose (Additional file 2: Table S1), and cumulative excretion of AD16 in feces accounted for 0.20% of the administered dose (Additional file 2: Table S2). In addition to protodrugs, oxidative metabolites can be detected in feces as well as metabolites that undergo sulfonation after oxidation.

### Safety and tolerability

Overall, there were 26 (41.9%), 11 (55.0%) and 5 (31.3%) subjects with one or more occurrences of treatment-emergent adverse event (TEAE) respectively in the SAD, MAD, and FE groups (Additional file 2: Table S3). Among them, 8 subjects (12.9%) in SAD and 4 (20.0%) in the MAD group presented Significant adverse events. All TEAEs in this experiment were judged to be possibly related, except the “left upper arm pain” in the MAD group. “Abnormal ECG” and “elevated triglyceride” were common TEAEs.

All TEAEs were graded as I/II, and all subjects who completed the follow-up showed recovery or improvement. No SAE occurred, and no subjects withdrew from the trial because of adverse reactions. In this study, tolerance-related adverse reactions were only observed in the SAD group. Out of the 62 subjects included in the SS set, 3 subjects had 3 tolerance-related adverse reactions of severity grade II following ingestion of the study drug, accounting for an incidence of 4.8%.

In the SAD arm, the AD16 and placebo groups exhibited about equal incidence of adverse events: 26 adverse events occurred in 19 subjects (39.6%) in the AD16 group, while 10 adverse events eventuated in 7 subjects (50.0%) in the placebo group.

In the AD16 group and the placebo group, the incidence of abnormal ECG T wave was 8.3% and 7.1%, respectively, and the incidence of elevated blood triglyceride was 14.6% and 14.3%, respectively (Additional file 2: Table S3).

In MAD, out of the 20 subjects included in the SS set, 15 TEAEs occurred in the 9 (56.3%) subjects from the AD16 group, and 3 TEAEs were observed in 2 subjects (50%) from the placebo group. Overall, AD16 demonstrated comparable safety to the placebo. In the AD16 group and the placebo group, the incidence of ECG Brugada wave was 6.3% and 0, respectively, and the incidence of elevated blood triglyceride was 12.5% and 25%, respectively. The incidence of TEAEs in 40 mg group was lower than that in 30 mg group (Additional file 2: Table S3).

In FE, among the 16 subjects included in the SS set, 2 subjects (12.5%) from the fasting group had 2 cases of TEAEs, and 3 subjects (18.8%) in the fed state had 3 TEAEs, showing similar TEAE incidence and severity in fasting state and postprandially. In the high-fat diet group and the fasting group, the incidence of abnormal ECG T wave was 12.5% and 6.3%, respectively, and the incidence of elevated blood triglycerides was 6.3% and 0 respectively (Additional file 2: Table S3).

## Discussion

The present study reports the first phase I clinical trial of AD16. Preclinical studies of AD16 in rats demonstrated its good oral bioavailability and blood–brain barrier penetration with a large safety window.

The linear dose analysis showed a *β* value slightly lower than 1, indicating an increase in blood concentration of AD16 with its increasing dose. Although approximately linear, the proportional increase in blood concentration was slightly lesser than the proportionate increase in dose. Moreover, their 90% CI is not entirely within the range of the judgment interval, and the linear dynamic properties cannot be determined. This may be because as the dose increases, AD16 is saturated with absorption, increasing blood concentration slightly less than the dose ratio.

The findings from trial studies administering multiple drug doses displayed that the trial drug doses were within the safety window and the effective dose could be maintained through the drug’s accumulation effect. Although the 40 mg dose exhibited higher exposure than 30 mg, the elimination and distribution characteristics were comparable in both groups. The efficacy and safety traits of these two dose groups could be further assessed in the follow-up therapeutic dose exploration test to support the therapeutic dose selection.

The results indicated that a high-fat diet decreases the rate, but not the extent, of AD16 absorption. Food can affect drug absorption through delayed gastric emptying, changes in gastrointestinal pH, and biliary excretion [23]. The delayed AD16 absorption may be ascribed to the high-fat diet-mediated slowing of gastric emptying and restraining the spread of the drug over the gastrointestinal wall. The fed and fasting state groups demonstrated analogous safety, elimination, and distribution, indicating that food did not affect the elimination and distribution of AD16. Similar to SAD, gastrointestinal side effects were not reported in the FE study. Overall, the high-fat diet significantly influenced the absorption rate of AD16. However, since the treatment of AD requires long-term medication, delayed absorption has little impact on the clinical application of AD16.

As for analyzing the excretion of AD16 metabolites in urine and feces, this study is the first clinical trial of AD16 tablets in humans, and the detection of metabolites is still being explored/implemented. Subsequent analysis will be supplemented according to the determined detection and analysis methods.

In the SAD study, the adverse events with high incidence in AD16 group and placebo group involved various examinations and various nervous system disease, but the incidence was similar in both groups. In the MAD study, the safety profile of the AD16 group was comparable to that of the placebo group. In the FE study, the overall incidence of adverse events was similar after a high-fat diet and in fasting state. The incidence of adverse events in the MAD and FE studies was comparable to that in the SAD group. All TEAEs were grades I and II, mostly grade I, and were recovered or improved except for 7 subjects whose outcome was unknown due to loss of follow-up. No dose-related toxic reactions were observed in all studies, and no SAE occurred. Therefore, it is concluded that AD16 is generally a safe and well-tolerated drug. However, due to the limited sample size, subsequent trials are warranted to explore its safety further.

There are some limitations in this study. Subjects were recruited from only one center in one country. The results might not represent the global and other regional populations. Second, because AD often requires long-term treatment, the results of the present study might not reflect the exact clinical scenario.

## Conclusions

The single and multiple doses of AD16 demonstrated favorable PK characteristics, good general safety, and well toleration in healthy Chinese male and female adults under a fed and fasted state. A fat-rich diet decreased the rate of AD16 absorption and lengthened the time to reach peak plasma concentration. The findings are promising to conduct further clinical studies on AD16 to determine its therapeutic role in AD.

### Supplementary Information

**Additional file 1.** CONSORT_checklist.

**Additional file 2:** **Table S1.** Cumulative urine excretion rate and renal clearance rate of oral 20 mg AD16 tablets. **Table S2. **Cumulative excretion rate of AD16 feces Rate in fecal samples after a single oral dose of AD16 tablets. **Table S3.** Summary of Adverse Events by System (SS).** Supplementary information S1. **Inclusion criteria and Exclusion criteria. **Supplementary information S2**. Randomization method (detailed).

## Data Availability

The datasets used and/or analyzed during the current study are available from the corresponding author upon reasonable request.

## Abbreviations

AD: Alzheimer’s disease
AUC: Area under the plasma concentration–time curve
AUC _0-∞_: Area under the plasma concentration–time curve from time zero to infinity
AUC_0-t_: Area under the plasma concentration–time curve from time zero to t
Aβ: Amyloid beta
CL/F: Apparent total clearance
*C*_max_: Maximum plasma drug concentration
*C*_min_: Minimum plasma drug concentration
*C*_avg__,ss_: Steady-state mean concentration
CTCAE: Common Terminology Criteria for Adverse Events
DF: Degree of fluctuation
ECG: Electrocardiogram
FE: Food effect
FES: Food effect analysis set
GMR: Geometric mean ratio
LC–MS/MS: Liquid chromatography-tandem mass spectrometry
MAD: Multiple ascending dose
MRT: Mean retention time
λz: Terminal disposition rate constant/terminal rate constant
MedDRA: Medical Dictionary for Regulatory Activities
*R*_ac_: Accumulation ratio
SAE: Serious adverse event
SAD: Single ascending dose
SOP: Standard operating procedure
SD: Standard deviation
*T*_max_: Time to reach peak plasma concertation
TEAE: Treatment-emergent adverse event
t_1/2_: Elimination half-life
Vd/F: Apparent volume of distribution
