# Supplementary material for: Safety, tolerability, pharmacokinetics and effects of diet on AD16, a novel neuroinflammatory inhibitor for Alzheimer’s disease: a randomized phase 1 study
Source: BMC Med. 2023 Nov 23;21:459. doi: 10.1186/s12916-023-03126-9 (PMC10666448; doi:10.1186/s12916-023-03126-9)
Supplement: Supplementary file 2 — Additional file 2: Table S1. Cumulative urine excretion rate and renal clearance rate of oral 20 mg AD16 tablets. Table S2. Cumulative excretion rate of AD16 feces Rate in fecal samples after a single oral dose of AD16 tablets. Table S3. Summary of Adverse Events by System (SS). Supplementary information S1. Inclusion criteria and Exclusion criteria. Supplementary information S2. Randomization method (detailed). [file 12916_2023_3126_MOESM2_ESM.doc]

**Supplementary**

**Table of Contents**

[Table S1. Cumulative urine excretion rate and renal clearance rate of oral 20mg AD16 tablets 2](#__RefHeading___Toc85814100)

[Table S2. Cumulative AD16 feces Rate in fecal samples after a single oral dose of AD16 tablets 3](#__RefHeading___Toc85814101)

[Table S3. Summary of Adverse Events by System (SS) 4](#__RefHeading___Toc85814102)-7

Supplementary information S1:[Inclusion criteria and Exclusion criteria 8](#__RefHeading___Toc85814111)-13

Supplementary information S2:Randomization method (detailed) [1](#__RefHeading___Toc85814111)4-15

**Table S1 Cumulative urine excretion rate and renal clearance rate of oral 20mg AD16 tablets**

|  | **Ae**  **(ng)** | **Fe_0-t_**  **(%)** | **CL_r_**  **(mL/h)** |
| --- | --- | --- | --- |
| N* | 8 | 8 | 8 |
| MEAN | 34944.55 | 0.17 | 9.06 |
| SD | 7946.39 | 0.039 | 2.74 |
| CV% | 22.74 | 22.60 | 30.23 |
| MIN | 19345.30 | 0.10 | 5.83 |
| Median | 37409.61 | 0.19 | 8.77 |
| MAX | 44480.05 | 0.22 | 14.62 |
| GMEAN | 33992.58 | 0.17 | 8.73 |
| CV%GMEAN | 26.97 | 26.79 | 29.23 |

Note：N*= Number of valid data;CV%GMEAN=sqrt(exp(variance of value after logarithmic conversion)-1)*100%.

Table S2 Cumulative AD16 feces Rate in fecal samples after a single oral dose of AD16 tablets

|  | **Ae**  **(ng)** | **Fe_0-t_**  **(%)** |
| --- | --- | --- |
| N* | 7 | 7 |
| MEAN | 39887.50 | 0.20 |
| SD | 26950.58 | 0.13 |
| CV% | 67.57 | 67.56 |
| MIN | 9536.57 | 0.05 |
| Median | 26602.76 | 0.13 |
| MAX | 76802.02 | 0.38 |
| GMEAN | 31243.34 | 0.16 |
| CV%GMEAN | 94.76 | 94.58 |

Note：N*= Number of valid data;CV%GMEAN=sqrt(exp(variance of value after logarithmic conversion)-1)*100%.

**Table S3 Summary of Adverse Events by System (SS)**

|  | **SAD** | | | | | | | | | | |  | **MAD** | | | | | |  | **FE** | | | |
| --- | --- | --- | --- | --- | --- | --- | --- | --- | --- | --- | --- | --- | --- | --- | --- | --- | --- | --- | --- | --- | --- | --- | --- |
| **AE terms** | **Placebo**  **N=14** | | **5mg**  **N=8** | **10mg**  **N=8** | **20mg**  **N=8** | **30mg**  **N=8** | **40mg**  **N=8** | **60mg**  **N=4** | **80mg**  **N=4** | **AD16**  **N=48** | |  | **Placebo**  **N=4** | | **30mg**  **N=8** | **40mg**  **N=8** | **AD16**  **N=16** | |  | **Fasted**  **N=16** | **Fed**  **N=16** | **AD16**  **N=16** | |
|  | **n（%）** | **m** | **n（%）** | **n（%）** | **n（%）** | **n（%）** | **n（%）** | **n（%）** | **n（%）** | **n（%）** | **m** |  | **n（%）** | **m** | **n（%）** | **n（%）** | **n（%）** | **m** |  | **n（%）** | **n（%）** | **n（%）** | **m** |
| Subjects with any AEs | 7  (50.0) | 10 | 3 (37.5) | 3 (37.5) | 3 (37.5) | 4 (50.0) | 3 (37.5) | 2 (50.0) | 1 (25.0) | 19 (39.6) | 26 |  | 2 ( 50.0) | 3 | 5 ( 62.5) | 4 ( 50.0) | 9 ( 56.3) | 15 |  | 2 (12.5) | 3 (18.8) | 5 (31.3) | 5 |
| Urinary tract infection | 1  (7.1) | 1 | 0 | 0 | 0 | 0 | 0 | 0 | 0 | 0 | 0 |  | 0 | 0 | 0 | 0 | 0 | 0 |  | 0 | 0 | 0 | 0 |
| Elevated white blood cell count | 0 | 0 | 0 | 0 | 0 | 0 | 0 | 0 | 1 (25.0) | 1  (2.1) | 1 |  | 0 | 0 | 0 | 0 | 0 | 0 |  | 0 | 0 | 0 | 0 |
| Urine white blood cell positive | 3  (21.4) | 3 | 0 | 1 (12.5) | 1 (12.5) | 0 | 0 | 0 | 1 (25.0) | 3  (6.3) | 3 |  | 1  ( 25.0) | 1 | 1 ( 12.5) | 0 | 1 ( 6.3) | 1 |  | 0 | 0 | 0 | 0 |
| Urine red blood cells positive | 1  (7.1) | 1 | 0 | 1 (12.5) | 0 | 1 (12.5) | 0 | 0 | 0 | 2  (4.2) | 2 |  | 0 | 0 | 3 ( 37.5) | 1 ( 12.5) | 4 ( 25.0) | 4 |  | 0 | 0 | 0 | 0 |
| Abnormal electrocardiogram | 1  (7.1) | 1 | 0 | 1 (12.5) | 1 (12.5) | 1 (12.5) | 1 (12.5) | 0 | 0 | 4  (8.3) | 4 |  | 0 | 0 | 0 | 1 ( 12.5) | 1 ( 6.3) | 1 |  | 1  (6.3) | 2 (12.5) | 3 (18.8) | 3 |
| Elevated blood triglycerides | 2  (14.3) | 2 | 0 | 1 (12.5) | 1 (12.5) | 2 (25.0) | 2 (25.0) | 1 (25.0) | 0 | 7 (14.6) | 7 |  | 1  ( 25.0) | 1 | 1 ( 12.5) | 1 ( 12.5) | 2 ( 12.5) | 2 |  | 0 | 1  (6.3) | 1  (6.3) | 1 |
| Elevated blood uric acid | 0 | 0 | 1 (12.5) | 0 | 0 | 0 | 0 | 0 | 0 | 1  (2.1) | 1 |  | 0 | 0 | 0 | 0 | 0 | 0 |  | 0 | 0 | 0 | 0 |
| Elevated blood lactate dehydrogenase | 0 | 0 | 0 | 0 | 0 | 1 (12.5) | 0 | 0 | 0 | 1  (2.1) | 1 |  | 0 | 0 | 0 | 0 | 0 | 0 |  | 0 | 0 | 0 | 0 |
| Decreased neutrophil count | 0 | 0 | 0 | 1 (12.5) | 1 (12.5) | 0 | 0 | 0 | 0 | 2  (4.2) | 2 |  | 0 | 0 | 0 | 0 | 0 | 0 |  | 0 | 0 | 0 | 0 |
| Increased total bile acids | 0 | 0 | 0 | 0 | 0 | 1 (12.5) | 0 | 1 (25.0) | 0 | 2  (4.2) | 2 |  | 0 | 0 | 1 ( 12.5) | 0 | 1 ( 6.3) | 1 |  | 0 | 0 | 0 | 0 |
| Urine acetone bodies | 0 | 0 | 0 | 0 | 0 | 0 | 0 | 0 | 0 | 0 | 0 |  | 0 | 0 | 1 ( 12.5) | 0 | 1 ( 6.3) | 1 |  | 0 | 0 | 0 | 0 |
| Elevated blood bilirubin | 0 | 0 | 0 | 0 | 0 | 0 | 0 | 0 | 0 | 0 | 0 |  | 0 | 0 | 0 | 1 ( 12.5) | 1 ( 6.3) | 1 |  | 0 | 0 | 0 | 0 |
| Headache | 1  (7.1) | 1 | 2 (25.0) | 0 | 0 | 0 | 0 | 0 | 0 | 2  (4.2) | 2 |  | 0 | 0 | 0 | 0 | 0 | 0 |  | 0 | 0 | 0 | 0 |
| Dizziness | 0 | 0 | 0 | 0 | 0 | 0 | 0 | 0 | 0 | 0 | 0 |  | 0 | 0 | 0 | 0 | 0 | 0 |  | 1  (6.3) | 0 | 1 (6.3) | 1 |
| Myalgia | 1  (7.1) | 1 | 0 | 0 | 0 | 0 | 0 | 0 | 0 | 0 | 0 |  | 0 | 0 | 0 | 0 | 0 | 0 |  | 0 | 0 | 0 | 0 |
| Limb pain | 0 | 0 | 0 | 0 | 0 | 0 | 0 | 0 | 0 | 0 | 0 |  | 0 | 0 | 1 ( 12.5) | 0 | 1 ( 6.3) | 1 |  | 0 | 0 | 0 | 0 |
| Anemia | 0 | 0 | 0 | 0 | 1 (12.5) | 0 | 0 | 0 | 0 | 1  (2.1) | 1 |  | 1 ( 25.0) | 1 | 0 | 1 ( 12.5) | 1 ( 6.3) | 1 |  | 0 | 0 | 0 | 0 |
| Nausea | 0 | 0 | 0 | 0 | 0 | 0 | 0 | 0 | 0 | 0 | 0 |  | 0 | 0 | 1 ( 12.5) | 0 | 1 ( 6.3) | 1 |  | 0 | 0 | 0 | 0 |
| Abdominal distension | 0 | 0 | 0 | 0 | 0 | 0 | 0 | 0 | 0 | 0 | 0 |  | 0 | 0 | 1 ( 12.5) | 0 | 1 ( 6.3) | 1 |  | 0 | 0 | 0 | 0 |

n=number of subjects

m = number of events

Electrocardiogram(ECG) abnormalities in SAD and FE are ECG T wave abnormalities, and ECG abnormalities in MAD are ECG Brugada waves

**Inclusion criteria and Exclusion criteria**

**SAD**

**Inclusion criteria**

1) Healthy subjects aged 18-45 years (including boundary values), male and female.

2) Weight ≥50kg (male) or ≥45kg (female) with a body mass index (BMI) of 19-24kg/m2(including the boundary values at both ends).

3) Have fully understood this study, participated voluntarily, and signed the Informed consent.

4) Subjects can communicate well with researchers and complete the study according to protocol.

5) Based on the results of physical examination, medical history, vital signs, electrocardiogram, chest X-ray, abdominal ultrasound, and laboratory examination, the subjects were considered to be in good health.

6) Subjects (including their partners) are willing to have no pregnancy plan for the next 30 days (female subjects) or 90 days (male subjects) and voluntarily take effective contraceptive measures.

**Exclusion criteria**

Subjects who meet 1 of the following exclusion criteria are excluded.

1) Hepatitis B surface antigen, hepatitis C antibody, syphilis antibody or HIV antibody positive.

2) Have any symptoms or related history of serious illness; Including but not limited to heart, liver and kidney diseases or other acute or chronic digestive tract and respiratory diseases, as well as diseases of the blood, endocrine, nervous, mental and other systems, or any other diseases or physiological conditions that can interfere with the results of the study.

3) A history of postural hypotension with frequent episodes.

4) A history of frequent nausea or vomiting due to any cause.

5) Any clear history of drug or food allergies, especially allergies to ingredients similar to the drugs in this study.

6) Have special dietary requirements and cannot comply with the uniform diet provided by the clinical research center.

7) A history of drug abuse or positive urine tests during the screening period.

8) The average daily smoking amount of ≥5 cigarettes in the 3 months before the test.

9) Alcoholics or regular drinkers in the 6 months prior to study screening, i.e. drinking more than 14 units of alcohol per week (1 unit of alcohol ≈360mL beer or 45mL spirits with 40% alcohol or 150mL wine), or positive breath tests for alcohol during the screening period.

10) Excessive consumption of tea, coffee (more than 6 cups) and/or caffeinated beverages (more than 1L) per day.

11) Surgical procedures, transfusions of blood or blood components within 1 month prior to screening.

12) Blood loss or donation of more than 400 mL in the 2 months prior to screening.

13) Participating in other clinical studies and taking trial drugs within 3 months prior to study screening.

14) who had received any medication in the 28 days prior to screening.

15) Pregnant and lactating women or women who had unprotected sex within 14 days.

16) Subjects who were unable to complete the study for other reasons or deemed unsuitable for inclusion by the researchers.

**MAD**

**Inclusion criteria**

1) Healthy subjects aged 18-45 years (including boundary values), male and female.

2) Weight ≥50kg (male) or ≥45kg (female) with a body mass index (BMI) of 19-24kg/m2

(including the boundary values at both ends).

1. Have fully understood this study, participated voluntarily, and signed the Informed consent.

4) Subjects can communicate well with researchers and complete the study according to protocol.

5) Based on the results of physical examination, medical history, vital signs, electrocardiogram, chest X-ray, abdominal ultrasound, and laboratory examination, the subjects were considered to be in good health.

6) Subjects (including their partners) are willing to have no pregnancy plan for the next 30 days (female subjects) or 90 days (male subjects) and voluntarily take effective contraceptive measures.

**Exclusion criteria**

Subjects who meet 1 of the following exclusion criteria are excluded.

1) Hepatitis B surface antigen, hepatitis C antibody, syphilis antibody or HIV antibody positive.

2) Have any symptoms or related history of serious illness; Including but not limited to heart, liver and kidney diseases or other acute or chronic digestive tract and respiratory diseases, as well as diseases of the blood, endocrine, nervous, mental and other systems, or any other diseases or physiological conditions that can interfere with the results of the study.

3) A history of postural hypotension with frequent episodes.

4) A history of frequent nausea or vomiting due to any cause.

5) Any clear history of drug or food allergies, especially allergies to ingredients similar to the drugs in this study.

6) Have special dietary requirements and cannot comply with the uniform diet provided by the clinical research center.

7) A history of drug abuse or positive urine tests during the screening period.

8) The average daily smoking amount of ≥5 cigarettes in the 3 months before the test.

9) Alcoholics or regular drinkers in the 6 months prior to study screening, i.e. drinking more than 14 units of alcohol per week (1 unit of alcohol ≈360mL beer or 45mL spirits with 40% alcohol or 150mL wine), or positive breath tests for alcohol during the screening period.

10) Excessive consumption of tea, coffee (more than 6 cups) and/or caffeinated beverages (more than 1L) per day.

11) Surgical procedures, transfusions of blood or blood components within 1 month prior to screening.

12) Blood loss or donation of more than 400 mL in the 2 months prior to screening.

13) Participating in other clinical studies and taking trial drugs within 3 months prior to study screening.

14) who had received any medication in the 28 days prior to screening.

15) Pregnant and lactating women or women who had unprotected sex within 14 days.

16) Subjects who were unable to complete the study for other reasons or deemed unsuitable for inclusion by the researchers.

**FE**

**Inclusion criteria**

Subjects must meet all inclusion criteria to be enrolled.

1) Healthy subjects aged 18-45 years (including boundary values), male and female.

2) Weight ≥50kg (male) or ≥45kg (female) with a body mass index (BMI) of 19-24kg/m2

(including the boundary values at both ends).

3)Have fully understood this study, participated voluntarily, and signed the Informed consent.

4) Subjects can communicate well with researchers and complete the study according to protocol.

5) Based on the results of physical examination, medical history, vital signs, electrocardiogram, chest X-ray, abdominal ultrasound, and laboratory examination, the subjects were considered to be in good health.

6) Subjects (including partners) are willing to have no pregnancy plans for the next 6 months and voluntarily use effective contraception.

**Exclusion criteria**

Subjects who meet 1 of the following exclusion criteria are excluded.

1) Hepatitis B surface antigen, hepatitis C antibody, syphilis antibody or HIV antibody positive.

2) Have any symptoms or related history of serious illness; Including but not limited to heart, liver and kidney diseases or other acute or chronic digestive tract and respiratory diseases, as well as diseases of the blood, endocrine, nervous, mental and other systems, or any other diseases or physiological conditions that can interfere with the results of the study.

3) A history of postural hypotension with frequent episodes.

4) A history of frequent nausea or vomiting due to any cause.

5) Any clear history of drug or food allergies, especially allergies to ingredients similar to the drugs in this study.

6) Have special dietary requirements and cannot comply with the uniform diet provided by the clinical research center.

7) A history of drug abuse or positive urine tests during the screening period.

8) The average daily smoking amount of ≥5 cigarettes in the 3 months before the test.

9) Alcoholics or regular drinkers in the 6 months prior to study screening, i.e. drinking more than 14 units of alcohol per week (1 unit of alcohol ≈360mL beer or 45mL spirits with 40% alcohol or 150mL wine), or positive breath tests for alcohol during the screening period.

10) Excessive consumption of tea, coffee (more than 6 cups) and/or caffeinated beverages (more than 1L) per day.

11) Ingest food or drink rich in xanthine, grapefruit or alcohol, caffeine (e.g., dragon fruit, mango, grapefruit, chocolate, coffee or tea) within 48 hours before administration.

12) Surgical procedures, transfusions of blood or blood components within 1 month prior to screening.

13) Blood loss or donation of more than 400 mL in the 2 months prior to screening.

14) Participating in other clinical studies and taking trial drugs within 3 months prior to study screening.

15) who had received any medication in the 28 days prior to screening.

16) Pregnant and lactating women or women who had unprotected sex within 14 days.

17) Subjects who were unable to complete the study for other reasons or deemed unsuitable for inclusion by the researchers.

Random method (detailed)

SAD:All subjects were assigned a unique digit screening number (Sxxx). After signing the informed consent, completing all screening evaluations, and confirming that the subjects met all enrollment criteria, they entered the clinical trial according to the order of completing screening evaluations, and were assigned the corresponding subject number, which was composed of 4 digits (xxxx) : the first and second digits represented the dose group.The last two are the random enrollment numbers of subjects in this dose group, and the random order is sorted according to the screening number of subjects from smallest to largest, such as 0101 representing the enrolled subject No. 01 of the first dose group, and so on.The drug number is consistent with the subject number, and a letter D is added before the subject number for differentiation.Randomization administrators unrelated to this study used a completely randomized method to generate subject numbers and drug numbers with SAS9.4 version of the PLAN process, a random seed for each dose group, and a random table of drugs for each dose group.The investigator or his designee shall sequentially extract the blinded drugs from the study drugs in the corresponding dose group according to the corresponding subject number.

MAD: The random method is the same as that of SAD group, but the number of subjects in MAD group is composed of MAD+4 digits (MADxxxx): the 1st and 2nd digits represent the number of dose groups, and the last 2 digits are the enrollment number of subjects in this dose group, which is sorted by subject screening number from small to large, such as MAD0101 generation Table 01 Enrolled subject in dose group 1

FE: A stratified (gender as a stratified factor) block randomization method was used to randomly assign group A (first cycle of fasting medication, second cycle of postprandial medication) or group B (first cycle of postprandial medication, second cycle of fasting medication) at a ratio of 1:1.The selected male subjects (8 cases) were assigned the random number R001 to R008 from small to large according to the size of screening number.The successful female subjects (8 cases) were assigned random numbers R009~R016 according to the size of the screening number, and random tables were generated using the PLAN process of SAS version 9.4.
